# Butanolic fraction of Moringa oleifera Lam. (Moringaceae) attenuates isoprotrenol-induced cardiac necrosis and oxidative stress in rats: an EPR study

**DOI:** 10.17179/excli2014-431

**Published:** 2015-01-15

**Authors:** Sunanda Panda

**Affiliations:** 1 School of Life Sciences, Devi Ahilya University, Vigyan bhavan, Khandwa Road, Indore 0452017, M.P. India

**Keywords:** Moringa oleifera, Quercetin, Isoproterenol, lipid peroxidation, EPR, cardiac markers

## Abstract

The preventive effect of *Moringa oleifera* polyphenolic fraction (MOPF) on cardiac damage was evaluated in isoproterenol (ISO) induced cardiotoxicity model of Wistar rats. Male rats in different groups were treated with MOPF orally at the dose of 50, 100 and 150 mg/kg/day for 28 days and were subsequently administered (s.c.) with ISO (85 mg/kg body weight) for the last two days. At the end of the experiment levels of serum troponin-T, creatine kinase-MB, lactate dehydrogenase, content of malondialdehyde (MDA), activities/levels of different cellular antioxidants were estimated in control and experimental groups. Additionally, scavenging potential to the hydroxyl radical of the fraction was measured by electron paramagnetic resonance (EPR). ISO administered rats showed significant increase in the levels of serum troponin-I, creatine kinase, lactate dehydrogenase, and heart tissue MDA content. Furthermore, marked reduction in the activities of antioxidants such as superoxide dismutase, catalase, glutathione peroxidase and reduced glutathione levels were observed. EPR study showed an increase in signal intensity in ISO-induced rats. Triphenyl tetrazolium chloride (TTC) staining of heart section revealed a marked increase in infarcted area in ISO-induced rats. Histological features of the heart also indicated a disruption in the structure of cardiac myofibrils in these animals. MOPF (100 mg/kg body weight) pretreatment prevented all these adverse effects of ISO. Present results show that the rich polyphenolic content of *Moringa oleifera* significantly reduced the myocardial damage and decreased the oxidative stress, possibly through hydroxyl radical scavenging activity as evidenced from the EPR spectra.

## Introduction

Cardiovascular diseases and their complications have been regarded as the leading causes of morbidity and mortality around the world, especially in developed countries. Although modern drugs are effective in treating this condition, they are mostly accompanied with adverse effects. 

The rat model of isoproterenol-induced myocardial necrosis, out of many well known models, has often been used to evaluate several cardiac dysfunctions (Rona et al., 1959[23], Panda et al., 2012[19]). Myocardial infarction, a highly prevalent ischemic condition characterized by tissue necrosis develops essentially due to an imbalance between oxygen need and actual supply (De Bono et al., 1992[3]), resulting in irreversible histopathological damages and subsequent cardiovascular complications (Gross, 2007[6]). The generation of highly cytotoxic free radicals through the auto-oxidation of catecholamines and a disturbance in the physiological balance between production of free radicals and an anti-oxidative defense system, have been implicated as important risk factors in the loss of integrity and function of myocardial membranes (Srivastava et al., 2007[28]).

In recent years, polyphenols have attracted considerable attention as agents that protect cells or molecules from oxidative myocardial injury. Epidemiological studies have suggested associations between the consumption of polyphenol-rich foods or beverages and the prevention of diseases. Recently, there has been renewed interest in medicinal plants and food products derived from them, which have been found to exhibit preventive measures in the treatment of cardiovascular disease (Panchal et al., 2011[18]). One such plant, *Moringa oleifera *Lam., known as drumstick tree in English, belongs to family Moringaceae. It is the most widely distributed species, especially in Asian countries and attributed with many pharmacological properties. Various parts of this plant such as the leaves, roots, seed, bark, fruit, flowers and immature pods are believed to act as cardiac and circulatory stimulants, possess antitumor, antipyretic, antiepileptic, anti-inflammatory, anti ulcer, antispasmodic, diuretic, antihypertensive, cholesterol lowering, antioxidant, antidiabetic, hepatoprotective, antibacterial and antifungal activities (Anwar et al., 2007[2]). An earlier study had shown that the crude extract of the leaves exhibit cardio protection (Nandave et al., 2009[16]). However, no effort has been made till to date to evaluate the antioxidant and cardioprotective effect in in vivo, using the phenolic fraction of leaves. Therefore, the present study was planned to evaluate the preventive effect of *Moringa oleifera* phenolic fraction (MOPF) on ISO-induced oxidative stress in rats. As the butanolic fraction of leaves is known to be rich in polyphenols, in the present study, this fraction was considered (Prasanth et al., 2011[20]). Identification of the polyphenols present in the butanolic fraction was also carried out by HPLC-analyses. 

Apart from evaluating the preventive effects of MOPF on ISO-induced myocardial necrosis in rats with reference to cardiac marker enzymes, biochemical and histopathological changes, for the first time an attempt was made to determine the potential of MOPF as a free radical scavenger using in vitro electron paramagnetic resonance spectroscopy (EPR) study. In addition to this, the free radical scavenging activities of the MOPF were measured in vitro by 2,2-diphenyl-1-picryl-hydrazyl (DPPH) assay.

## Materials and Methods

### Chemicals

Activities of CK-MB (Creatine kinas-MB), LDH(Lactate dehydrogenase) and SGPT (serum glutamic-pyruvic transaminase) were measured in the serum using standard commercial kits. The level of cardiac troponin-T (cTnT) in the serum was quantitatively measured by means of a highly specific enzyme immunoassay using commercially available kits. Ellagic acid, kaempferol, quercetin and rutin, isoproterenol and spin trap, 5,5'-dimethyl-1-pyroline-N-oxide (DMPO), DPPH were purchased from Sigma and Aldrich (St. Louis, MO). Other chemicals and solvents were purchased from Merck chemicals Mumbai, India.

### Preparation of plant extract

Fresh, healthy leaves of *M. oleifera* were collected from departmental botanical garden, authenticated before using in the experiment and a voucher specimen (No. MO/05-09) was deposited in the departmental herbarium for further reference. Powdered material (100 g) was extracted with 70 % (v/v) ethanol at room temperature for 3 days. The yield of the extract was (20.1 %) in weight by weight (w/w). The procedure for solvent-solvent separation was adopted from Toma et al. (2012[29]) .The butanol fraction was prepared by successive extraction steps starting from crude ethanol extract using n-hexane, dichloromethane and butanol. After completing the separation process, the solvents were recovered by rota vapor. The separates were dried in steam bath at 40 °C and kept in the refrigerator for the experiments. The yields of n-hexane, dichloromethane, and n- butanol were 1.1 %, 0.9 %, and 1.9 % (w/w), respectively.

### HPLC analysis 

The major phenolic compounds in butanolic fraction and its quantification was done using a Jasco HPLC system fitted with reverse phase C-18 coloumn (250 x 4 mm). The mobile phase consisted of methanol and water (7:3). The flow rate, 1.0 ml/min, injection volume 20 µl, and the monitoring wavelength, 254 nm were maintained. Results (mg/g dry wt.) were obtained by comparison of peak areas of the samples with that of standards.

### Animals

Male Wistar rats, weighing 160–170 g, were used in the study. They were kept in the departmental animal house under controlled conditions of temperature at 25 ± 2 °C, and light:dark cycle of 12 h each with the provision of laboratory feed and water ad libitum. The study was conducted in accordance with the protocol approved by the Committee for the Purpose of Control and Supervision of Experiments on Animals, (CPSEA) New Delhi, India and the Institutional Animal Ethics Committee (779/2013-14).

### Experimental design 

Thirty five healthy rats were divided into five groups of seven each. Group I animals receiving distilled water (0.1 ml/day/animal) served as control, whereas those of group II received only vehicle and ISO 85 mg/kg, subcutaneously (s.c), for last 2 consecutive days to induce myocardial injury (Karthikeyan et al.,2007[9]). Animals of group III, IV and V were administered with the extract at the dose of 50, 100 and 150 mg/kg/ day respectively for 28 days. 

### Biochemical estimations 

On the day of termination, overnight fasted animals were sacrificed under mild anesthesia, blood from each animal was collected and serum was separated for the estimation of different biochemical indices, including cardiac cTnT, CK-MB, SGOT, SGPT and LDH. After exsanguinations, heart tissues were removed quickly, washed with phosphate buffered saline (PBS, pH 7.4). The tissue homogenate was then centrifuged at 10,000 g for 30 min at 4 °C and the supernatant was used for the biochemical estimations. Malondialdehyde (MDA) levels, an index of lipid peroxidation were estimated by concentration of thiobarbituric acid reactive substances (TBARS) in heart tissues as followed by Ohkawa et al. (1979[17]), SOD, CAT, GPx, and GSH were assayed with the methods of Marklund and Marklund (1974[13]), Aebi (1983[1]), Rotruck et al. (1973[24]) and Ellman (1959[4]) respectively. Protein was estimated according to Lowry et al. (1951[11]) using BSA as standard to calculate the protein content of the samples. In addition to this, in vitro free radical scavenging effect of MOPF on DPPH was also evaluated by the method of Mensor et al. (2001[14]).

### Electro paramagnetic resonance (EPR) analyses

EPR analyses of cardiac tissues of different groups of animals against hydroxyl radical were measured using an EPR spectrometer (JES-FA; Jeol, Japan). Instrument was set with microwave power of 8.0 mW, magnetic field of 337.98 ± 5 mT, modulation width 0.1 mT, amplitude 50, modulation frequency 100 KHz, time constant 0.1 s, and sweep time 2 min. Fenton reaction system was employed to generate hydroxyl radicals (OH.) which were spin-trapped by DMPO, as done earlier (Panda et al., 2012[19]). Quantification of the DMPO signal intensity was performed by comparing the amplitude of the observed signal to a standard Mn^2+^/MgO marker.

### TTC staining

TTC (Triphenyl tetrazolium chloride) assay was performed according to method of Lie et al. (1975[10]) to determine the myocardial necrotic area. In brief, the heart was transversely cut across the left ventricle, and sections of 2 mm to 3 mm thick were incubated in 1 % TTC solution prepared in phosphate buffer (pH 7.4) for 30 min at 37 °C for differentiation of viable tissue from necrotic areas.

### Histopathological studies

Myocardial tissue after removal was immediately fixed in 10 % buffered neutral formalin solution. After fixation was complete, tissues were embedded in paraffin and serial sections were cut. Each section was stained with hematoxylin and eosin. The sections were examined under light microscope and photomicrographs were taken.

### In vitro free radical scavenging action of MOPF

The antioxidant activity was measured in terms of hydrogen donating or radical scavenging ability using the stable radical DPPH. The ability of MOPF to scavenging the stable free radical, DPPH was measured as a decrease in absorbance at 517 nm (Mensor et al., 2001[14]; Prince et al., 2010[21]). Briefly, 1 ml of 0.3 mM of DPPH solution was added to 1 ml each concentration of the test solution, and was incubated in the dark at room temperature for 30 min. The change in colour from deep violet to light yellow was then measured at 517 nm on a spectrophotometer. 

The percentage scavenging of DPPH• by MOPF was calculated as follows:

% Scavenging of DPPH•= (Control OD - Test OD) / Control OD) × 100

### Statistical analysis 

Data are presented as mean ± S.E.M and were analyzed by one-way ANOVA, with post-hoc comparisons by Student Newman-Keuls test using a Prism version 4 for Windows (Graph Pad, San Diego, CA). P < 0.05 was considered as significant.

## Results

### HPLC analyses

**Figure 1:**
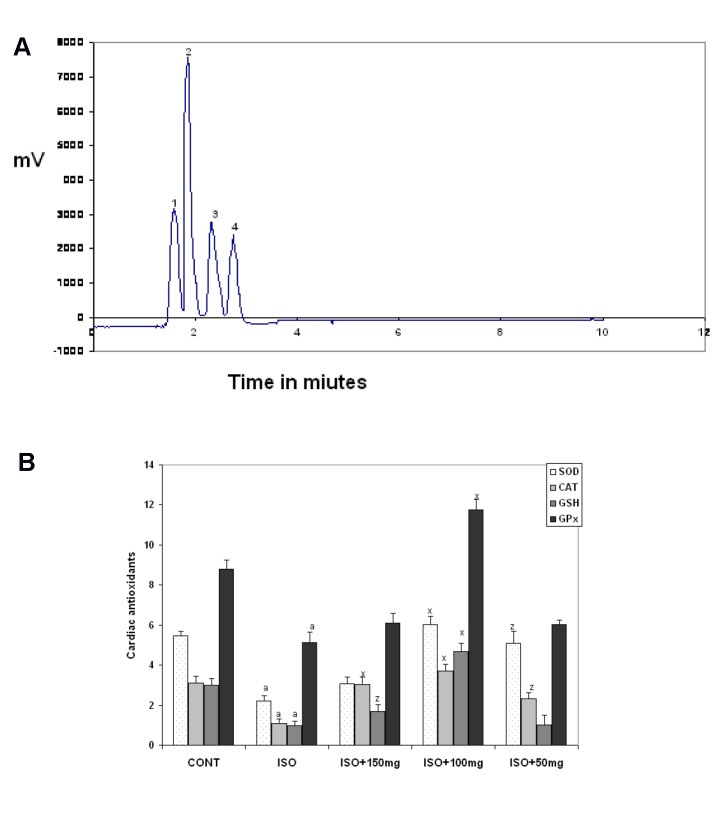
A) HPLC chromatogram of butanolic fraction of *M. oleifera* leaves (MOPF). Peak 1: Rutin; Peak 2: Quercetin; Peak 3: Kaempferol and Peak 4: ellagic acid Notes: The mobile phase consisted of methanol and water (7:3), with the flow rate, 1.0 ml/min; Column: Hypersil ODS C18 (250 x 4mm). Detector: DAD, 254 nm; Injection volume 20 µl. B) Effect of *M. oleifera *polyphenolic fraction (MOPF) on isoproterenol(ISO)-induced changes in myocardial catalase (CAT; µM H_2_O_2_ decomposed/min/mg protein X10), superoxide dismutase (SOD, U /mg protein) and glutathione peroxidase (GPx, U/mg protein) and GSH (µM GSH/mg protein) levels. Control animals received 0.1 ml distilled water/d/animal, ISO at 85 mg/kg for last 2 consecutive days and MOPF at 50, 100 and 150 mg/kg/d for 28 days pretreated to ISO-induced rats. Each vertical bar represents the mean ± SEM (n=7). ^a^, *P* < 0.001, as compared to the respective control value; whereas ^x^, *P* < 0.001 and ^z^, *P* < 0.05 as compared to respective value of ISO–induced group.

Using HPLC the presence of rutin (370 µg/g), quercetin (980.16 µg/g), kaempferol (490.5 µg/g) and ellagic acid (120.1 µg/g) was revealed. In the butanolic fraction quercetin was most abundant (Figure 1a, Peak 2(Fig. 1)).

### Cardiac lipid peroxidation and antioxidants

Following ISO administration, a marked increase in MDA level with a concomitant decrease in endogenous antioxidants such as SOD, CAT, GPx, and GSH levels were observed when compared to the control value (Table 1(Tab. 1) and Figure 1b(Fig. 1)). A significant reduction in MDA levels was observed in MOPF pretreated (50,100 and 150 mg/kg) ISO-induced rats when compared to group treated with ISO (Table 1(Tab. 1)). Pretreatment with MOPF (100 mg/kg) increased SOD, CAT, GPx and total GSH whereas MOPF at dose of 50 mg/kg/day could only increase the activities of the CAT and SOD. The dose 150 mg/kg/day increased only CAT and GSH level significantly.

### Cardiac markers

As shown in Table 1(Tab. 1), treatment of rats with ISO significantly increased the activity/levels of LDH, CK-MB and cTnT in the serum as compared to control animals. While no significant changes were observed by 150 mg/kg, MOPF at 50 and 100 mg/kg could decrease significantly all the activities/levels of aforesaid parameters when compared to ISO-control animals.

### ·OH scavenging action of MOPF by EPR study

**Figure 2:**
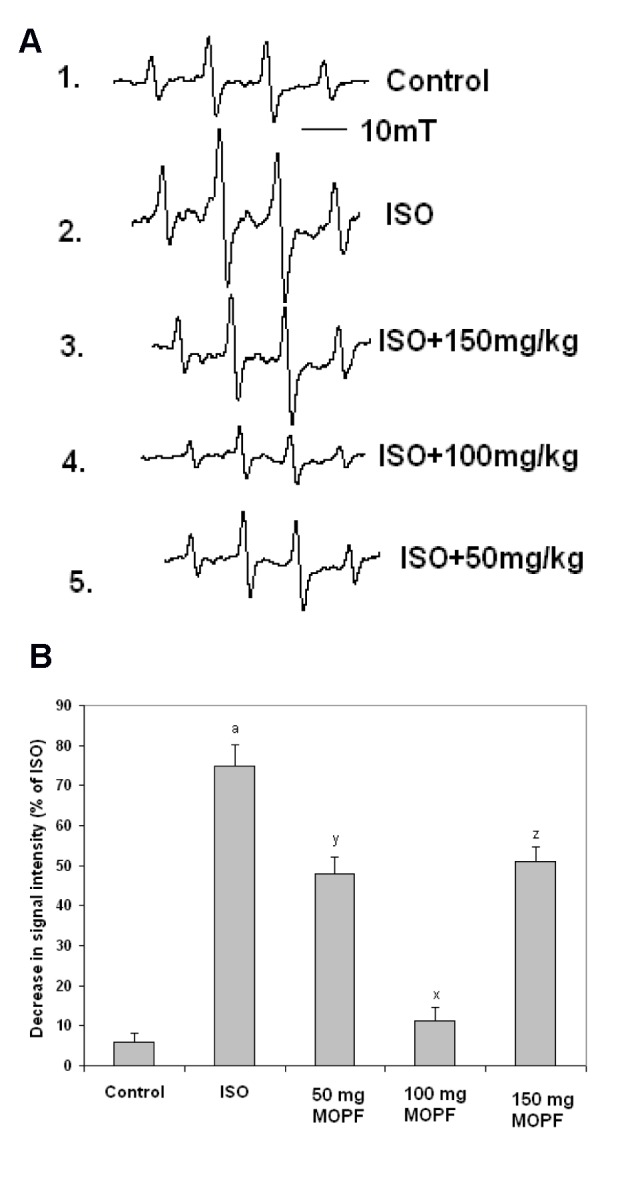
A) Different EPR signals, as recorded in the presence of the reaction mixtures of H_2_O_2_ (1 mM), FeSO4 (0.2 mM) and DMPO (0.4 mM). 1: control; 2: ISO; 3: ISO + MOPF 150 mg/kg; 4: ISO + MOPF 100 mg /kg and 5. for ISO + MOPF 50 mg/kg. B) Percent decrease in DMPO-OH signal intensities (calculated from the value of ISO-treated group) in the presence of ISO + MOPF (50 mg/kg); ISO + MOPF (100 mg/kg) and ISO + MOPF (150 mg /kg). Statistical comparisons were performed by one-way ANOVA. ***P < 0.001 and **P < 0.01 as compared to ISO control value (n=5).

Administration of ISO under ex vivo condition led to an increased formation of free radicals (FRs) in heart tissues, which were depleted by MOPF treatment to a greater extent. MOPF inhibited the DMPO-OH signal height in a concentration-dependent manner (Figure 2a(Fig. 2)). DMPO-OH signal intensities were reduced to 51, 75 and 31 % in the heart tissues at the doses of 50, 100 and 150 mg/kg of MOPF pretreated animals respectively, which were ISO-induced indicating a greater reduction in the 100 mg/kg group (Figure 2b(Fig. 2)).

### TTC staining

**Figure 3:**
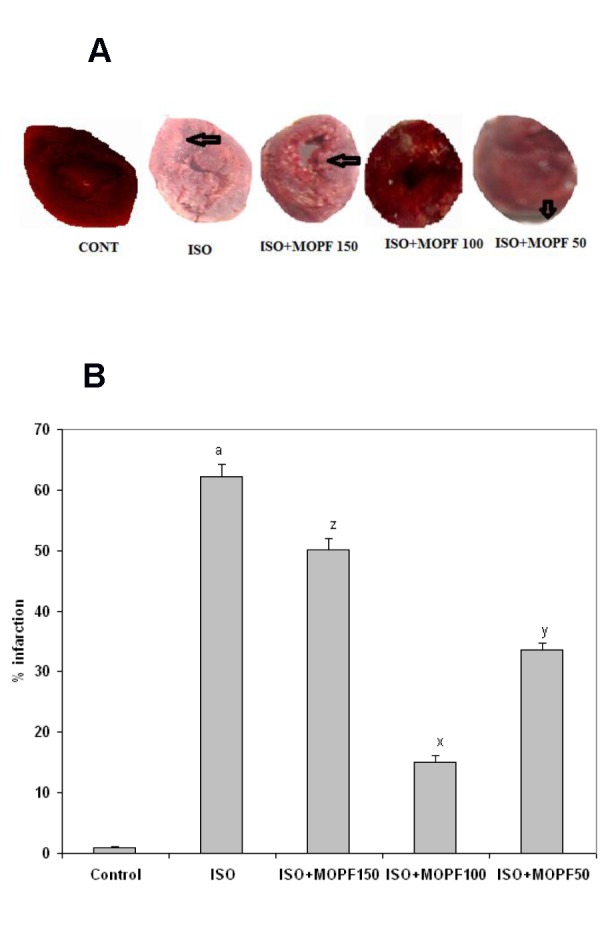
A) Triphenyl tetrazolium chloride (TTC) stained rat heart of different experimental animals. Normal control heart shows completely viable myocardial tissue (LDH enzyme active) without infarction, while ISO-treated (85 mg/kg) rat heart shows more infracted area, that did not stain with TTC because of enormous LDH enzyme leakage. Normal rat pretreated with MOPF (150 mg/kg) shows heart tissue stained positively for viability with mild LDH enzyme leakage. Pretreatment with MOPF (100 mg/kg) in ISO-induced rats showing some what similar stain to that of normal control rats (LDH enzyme active) and ISO-induced rats pretreated with MOPF (50 mg/kg) show heart tissues, stained positively for viability (mild LDH enzyme leakage). B) Calculated area (in %) of necrosis following the treatment of MOPF at three different doses (50, 100 and 150 mg/kg) for 28 days. ^a^*P* < 0.001 as compared to the respective value of control animals. ^x^*P* < 0.001, ^y^*P* < 0.01 and ^z^*P* < 0.001 as compared to the respective value of the ISO induced animals.

Detection of myocardial necrosis by TTC staining revealed that in ISO alone group, a large unstained area with more necrotic patches exists indicating a significant leakage of LDH as compared to control. In the control group, viable myocardial tissue was observed as evident by the formation of red formazan with LDH of the myocardial tissue. Similarly, animals pretreated with MOPF (100 mg/kg) showed major portion of heart tissue stained positively for viability with little LDH enzyme leakage and less necrotic area (Figure 3a(Fig. 3)). Figure 3b(Fig. 3) indicates the increased infarction area in ISO-intoxicated group (62.2 %), which was reduced to 15.1 % with the treatment of MOPF.

### Effect on histopathological changes in rat myocardium

**Figure 4:**
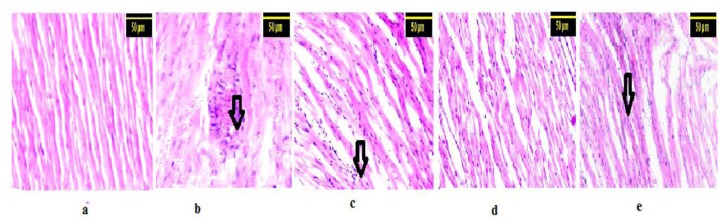
Histopathological analysis of rat myocardium: a: The rat heart from control group showing normal appearance of cardiac fibers. b: The rat heart of ISO alone group showing massive disruption and fragmentation of the myofibril structure and lymphatic infiltrations. c: The rat heart of MOPF (150 mg/kg) + ISO group, showing marked infiltration of inflammatory cells. d: Rat heart treated with ISO+ MOPF (100 mg/kg), showing normal architecture of heart. e: MOPF (50 mg/kg) along with ISO group is showing less infiltration of inflammatory cells. Arrow indicates cellular infiltrations of inflammatory cells (H X E 100 x).

The histoarchitecture of the cardiac tissue of control rats appeared to be normal. ISO-induced rats exhibited extensive myofibrillar degeneration, marked inflammatory infiltration of leucocytes, edema, necrosis and loss of striations. MOPF (150 and 50 mg/kg) revealed lesser degree of necrosis and less inflammatory infiltrate in to the myocardium (Figure 4(Fig. 4)). In MOPF (100 mg/kg) pretreated rats which received ISO showed nearly normal myofibrillar structure with mild degenerative changes and no inflammatory infiltrate in to the myocardium.

### Effect of MOPF in in vitro free radical (DPPH•) scavenging activity

**Figure 5:**
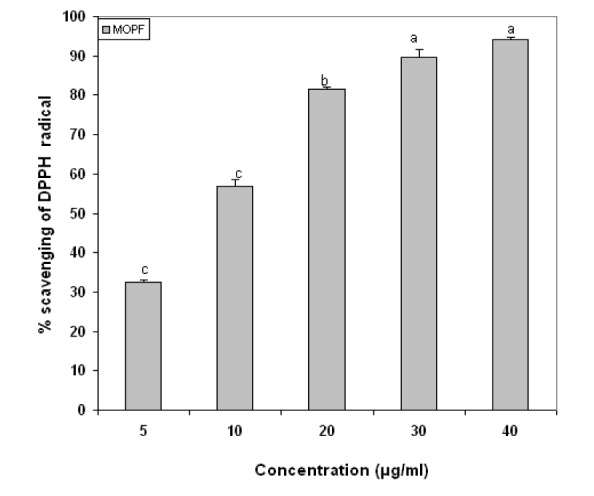
In vitro 1,1-diphenyl-2-picrylhydrazyl (DPPH) radical scavenging effect of *Moringa oleifera* butanolic fraction. DPPH solution was added to the test solutions of MOPF at concentrations of 5 to 40 µg/ml and was incubated in the dark at room temperature. The change in color from deep violet to light yellow was then measured at 517 nm on a spectrophotometer. Finally expressed in % scavenging of DPPH. Each vertical bar represents the mean ± SEM (n=3). Proportionate increase in % scavenging is observed with increase in MOPF concentrations.

Figure 5(Fig. 5) shows the percentage scavenging effects of MOPF on the free radical, DPPH•. The percentage scavenging effects of MOPF on DPPH• at different concentrations 5, 10, 20, 30 and 40 µg/ml were found to be 32.46, 56.99, 82.66, 91.66 and 91.46 % respectively. A significant dose dependent inhibition of DPPH activity was observed by MOPF with an IC_50_ value of 19.92 ± 1.19 µg/ml, whereas for quercetin, it was 19.95 ± 1.17 µg/ml.

## Discussion

Isoproterenol is believed to induce cardiotoxicity in the form of myocardial infarction (MI) in animals as a result of disturbance in physiological balance between the production of free radicals and antioxidant defense system (Zhou et al., 2008[31]). In fact, generation of highly cytotoxic free radicals through auto-oxidation of catecholamines has been implicated as one of the important causative factors in ISO-induced myocardial damage (Singal et al., 1982[26]).

The present finding revealed marked elevation of cardiac markers such as CK-MB, LDH and cTnT in the serum of ISO- induced rats which are in line with the earlier reports (Mair, 1999[12]; Radhiga et al., 2012[22]). The increase in the activities of CK-MB, LDH and level of cTnT in serum were in ISO-induced rats, may be due to the leakage from the heart as a result of necrosis induced by ISO ( Radhiga et al., 2012[22]). Interestingly, administration of 100 mg/kg of test compound, MOPF lowered significantly the activity of CK-MB, LDH and decreased the levels of cTnT, which could be due to its action on maintaining membrane integrity and thereby restricting the leakage of these cardiac markers into circulation.

Lipid peroxidation is an important pathogenic event in myocardial necrosis (Halliwell and Chirico, 1993[7]). Rats treated with ISO showed significant increase in the levels of MDA in the heart tissue as compared to normal control rats. The increased levels of lipid peroxides in ISO-induced animals might be due to free radical mediated membrane damage. Interestingly, all the three doses of MOPF were able to decrease the MDA level significantly, suggesting the LPO inhibitory effects of MOPF. The earlier reports also demonstrated that *M. oleifera* leaves protected the tissues against oxidative stress in rats (Verma et al., 2009[30]; Sreelatha and Padma, 2011[27]). Oxidative stress has been shown to play an important role in the pathophysiology of ischemic heart disease. Oxygen radicals can produce deleterious effects on the myocardium, including contractile dysfunction and structural damage (Josephson et al., 1991[8]). In this study, SOD activity decreased significantly in the ISO induced rats, which might be due to an excessive formation of superoxide anions. A decrease in SOD activity can result in the decreased removal of superoxide anions which can be harmful to the myocardium (Sharma et al., 2001[25]). The activities of CAT and GPx were also declined significantly due to an inactivation of the H_2_O_2_ scavenging enzymes. Glutathione is an important antioxidant which plays the role of an intracellular radical scavenger and is a substrate for many xenobiotic elimination reactions. The increased levels of GSH observed in MOPF pretreated rats resulted in increased activity of GPx in ISO-induced rats. Decreased levels of lipid peroxidation in MOPF pretreated ISO-induced rats improved the antioxidant system. Thus, the antioxidant property of MOPF appears to protect the myocardial tissue against ISO-induced oxidative damage.

The present study indicated, for the first time, that MOPF could directly quench ·OH radical and exert a protective effect against ISO-induced cardiac injury. EPR studies also demonstrated that the signal height of DMPO-OH, proportional to the amount of ·OH, was decreased (about 85 %) in the presence of MOPF (100 mg/kg) which indicated that this dose effectively suppressed the formation of the DMPO-OH adduct. Therefore, MOPF has an ability to directly scavenge ·OH radicals. This is in accordance with the recent observation that this dose can inhibit lipid peroxidation to a greater extent (82.5 %) as compared to two other doses (61 % for 50 mg/kg and 36 % for 150 mg/ kg). The decrease in signal intensity following the administration of the test extract, confirmed the view that butanolic fraction rich in quercetin protects the cardiac tissues by its direct free radical scavenging actions.

In ISO-induced rats major portion of the heart was found to be infarcted (appearance of patches of pale white color) that did not stain with triphenyl tetrazolium chloride. Interestingly, MOPF (100 mg/kg) depicted heart tissue stained positively for viability (mild LDH enzyme leakage) and reduced necrosis to a greater extent, which might be due to its potent antioxidant activity which prevents from damage and leakage of LDH.

While in ISO-induced rats large unstained region with more necrotic patches (62.2 %) were observed, the ISO treated animal that received the MOPF fraction at 100 mg/kg exhibited major portion stained positively showing tissue viability with less necrosis (15.1 %).

On histopathological examination, the ISO-induced myocardium showed marked necrosis, degeneration and disruption of cardiac myofibres and infiltration of inflammatory cells. Pretreatment with MOPF (100 mg/kg) afforded maximal protection as compared to other two doses and showed normal cardiac fibers and less lymphocytic infiltration as seen with the ISO treated group, further supporting the cardio-protective potential of MOPF.

As in MOPF pretreated ISO-induced rats, myocardium showed normal cardiac fibers, it appears MOPF does not possess any adverse effects, rather protects the myocardial tissues from damage.

In the present in vitro investigation MOPF also showed appreciable antioxidant activity on the DPPH scavenging effect. The DPPH. radical has been used widely in the model system to investigate the scavenging activities of several antioxidant substances (Gregus et al., 1996[5]). DPPH. radical is scavenged by antioxidants through the donation of hydrogen, forming the reduced DPPH-H•. The results in the present study also showed that the MOPF fraction significantly reduced DPPH radicals. The highest percentage of DPPH• scavenging effect of MOPF was found to be 91.66 %, at the concentration of 40 µg/ml. These findings do support the free radical scavenging activities of the test fraction. Thus, from the results of in vitro study, it is clear that the excessive free radicals produced by ISO are effectively quenched by MOPF. 

In butanolic fraction the major component was found to be quercetin and previous study had shown that quercetin scavenges ·-O2 and ·OH effectively protecting the cardiomyoctes from the cardiotoxic action of ISO (Prince et al., 2010[21]). Data on the quercetin content in Moringa leaves were reported to be 232.5 mg/kg of dry weight (Miean and Mohamed, 2001[15]). Most likely the cardio protection in butanolic fraction of the test drug is mediated through its major compound quercetin. 

## Conclusion

It can be concluded that antioxidant and free radical scavenging property of MOPF seem to protect the myocardium against ISO-induced oxidative damage. Although earlier the cardio protective effect of crude extract was reported, the possible constituents responsible for the cardioprotection have not been established yet. It appears that presence of polyphenols such as quercetin, kaempferol, rutin, and ellagic acid, primarily the former one, in butanolic fraction impart free radical scavenging and antioxidant effects, thereby protecting the myocardium against ISO-induced cardiac damage. Protection of myocardium by butanolic fraction of *Moringa oleifera* is likely due to the detoxification of isoproterenol through antioxidant defense system. Further studies are required to find out the exact mechanism of action of MOPF. 

## Acknowledgements

This work was supported by the grant received from Department of Science and Technology (DST), New Delhi, India under (WOS-A) scheme to Dr. Sunanda Panda.

## Conflict of interest

No conflict of interest to disclose.

## Figures and Tables

**Table 1:**
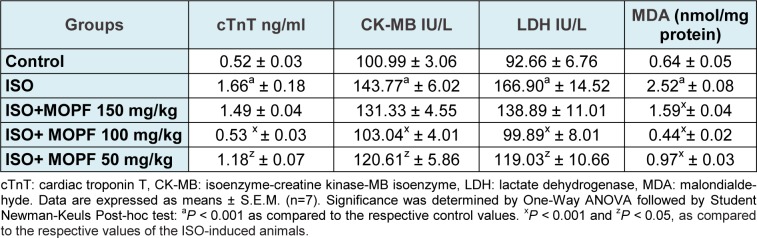
Effect of MOPF fraction on the activities/levels of cTnT, CK-MB, LDH and MDA level in control and MOPF (150 mg/kg, 100 mg/kg and 50 mg/kg) pretreated ISO-induced rats
